# Antimicrobial Use on 36 Beef Feedlots in Western Canada: 2008–2012

**DOI:** 10.3389/fvets.2019.00329

**Published:** 2019-10-17

**Authors:** Stephanie A. Brault, Sherry J. Hannon, Sheryl P. Gow, Brian N. Warr, Jessica Withell, Jiming Song, Christina M. Williams, Simon J. G. Otto, Calvin W. Booker, Paul S. Morley

**Affiliations:** ^1^Department of Clinical Sciences, College of Veterinary Medicine & Biological Sciences, State University, Fort Collins, CO, United States; ^2^Feedlot Health Management Services Ltd., Okotoks, AB, Canada; ^3^Canadian Integrated Program for Antimicrobial Resistance Surveillance, Public Health Agency Canada, Saskatoon, SK, Canada; ^4^School of Public Health, University of Alberta, Edmonton, AB, Canada; ^5^Veterinary Education, Research and Outreach Center, Texas A&M University and West Texas A&M University, Canyon, TX, United States

**Keywords:** antimicrobial use, feedlot cattle, bovine respiratory disease, Canada, quantification

## Abstract

The accurate quantification of antimicrobial use (AMU) in production animals is critical for monitoring trends in exposure to antimicrobial drugs (AMD) over time and examining potential associations with antimicrobial resistance in bacteria. In this study, a census sample of cattle was used to quantify individually-dosed and in-feed AMU as both numbers of animal daily doses (nADD) and total grams of AMD (gAMD) used in cattle placed in 36 western Canadian feedlots between 1-November, 2008 and 31-October, 2012; representing about 21.5% of fed cattle in Canada during that time period. Of the ~2.6 million cattle placed during the 48-month period, 45% were calves, 63% were male, 62% arrived in the fall or winter, and 39% were assessed as high risk for developing bovine respiratory disease (BRD). The proportion of cattle categorized as high risk (HR) for developing BRD was consistent over the 4 years of placement cohorts. Both medically important AMU and ionophore use were summarized but presented separately. A decrease in AMU was observed over the study period, both as nADD and total gAMD, which was primarily driven by a decline in the in-feed administration of tetracyclines. Most in-feed AMU was directed toward prevention and control of liver abscesses. The majority of individually dosed AMU was administered as metaphylaxis to address BRD risks, with category III AMD (medium importance to human medicine as categorized by Health Canada Veterinary Drugs Directorate) used most frequently. Not surprisingly, risk level for developing BRD influenced parenteral AMD exposures, with 95% of cattle categorized as being HR for developing BRD receiving individually dosed AMD compared to 59% of cattle categorized as being low risk (LR) for developing BRD. Cattle categorized as HR for developing BRD were more likely to receive macrolides for BRD metaphylaxis compared to cattle categorized as LR for developing BRD, and cattle categorized as LR for developing BRD were more likely to receive tetracycline for the same purpose. In summary, these data provide an unprecedented representation of AMU in fed cattle in western Canada and direction for future monitoring of AMU in fed cattle.

## Introduction

Antimicrobial drugs (AMD) are important tools for maintaining human and animal health. In North America, AMD are widely used to support feedlot cattle health. Of feedlots with more than 1,000 head of cattle in the United States, 87.5% administered AMD to animals by injection or in feed or water (1). There are concerns that widespread antimicrobial use (AMU) is an important driver of selection for antimicrobial resistance (AMR), which may be threatening the ongoing effectiveness of AMD to combat disease in people and animals (2). While AMU in any context has the potential to select for AMR, use in agricultural animals has been under increasing scrutiny due to the potential risk of transmitting resistant bacteria from animals to people by direct contact, environmental contamination, and/or consumption of contaminated meat (3–5). Because of these concerns, the concept of antimicrobial stewardship has risen to the forefront of discourse in veterinary medicine. Antimicrobial stewardship in veterinary medicine, as defined by the American Veterinary Medical Association (AVMA), “includes providing systems of care to reduce the incidence of common diseases, making evidence-based decisions about the use of AMD, and using AMD judiciously, with ongoing evaluation of the outcomes of use and consideration for animal caretakers' available resources (6).” Recognizing the particular need to preserve the effectiveness of those AMD relevant to human medicine, the World Health Organization (WHO) has published guidelines presenting evidence-based recommendations and best practice statements on the use in food animals of “medically important antimicrobials,” defined as antimicrobial classes used in human medicine. Medically important antimicrobials are further categorized by the WHO according to specified criteria as “important,” “highly important,” or “critically important” for human medicine. The WHO recommends that the overall use of medically important AMD in food animals be reduced, with complete restriction of their use for growth promotion and in animals in which clinical disease has not been diagnosed. The WHO further suggests that critically important AMD should only be used for treatment of individual sick animals, and that highest priority critically important AMD should not be used in food animals (7). Similarly, Health Canada classifies AMD in categories I to IV based, on importance to human health (8); category I are very high importance, category II are high importance, category III are medium importance, and category IV have low importance related to public health.

Bovine respiratory disease (BRD) is the most common cause of morbidity and mortality in beef cattle, and a frequent reason for antimicrobial use (9). The microbes implicated in BRD are largely ubiquitous in cattle populations, and most of the bacterial organisms, e.g., *Mannheimia haemolytica, Pasteurella multocida*, and *Histophilus somni*, are normal inhabitants of the bovine upper respiratory tract (10). The likelihood of manifestation of disease in cattle is influenced by numerous factors, including host and environmental determinants, pathogen characteristics, and management practices. Cattle recently arrived at the feedlot are particularly susceptible to developing BRD. They are stressed by the transportation event, unaccustomed to their new environment, and often have been recently weaned, all of which compromise immunity. In addition, these cattle have often not been previously vaccinated against BRD pathogens, and there is frequently extensive commingling of cattle from different sources leading to exposure to infectious diseases (11).

Diagnosis in individual cattle and therefore targeted individual treatment is hampered by the tendency of cattle as prey animals to mask signs of disease and the lack of rapid, sensitive, and specific disease identification methods (12). Therefore, risk assessment for BRD is a critical component of commercial feedlot production. Risk assessment for BRD is typically done on each group of feeder cattle purchased, and the result of the BRD risk assessment is the BRD risk assignment. This assignment is based on algorithms that include factors such as age class (calf vs. yearling), body weight (often a proxy for age), procurement method (sale barn vs. ranch direct), amount of commingling before and after arrival, and previous vaccination and management history. For each assigned BRD risk category, feedlot veterinarians develop the most appropriate program for mitigating the risk of BRD. Each program includes a variety of components, including vaccination and revaccination, on-arrival antimicrobial use, biosecurity procedures, disease detection and treatment, animal husbandry practices, feeds and feeding programs, and monitoring and animal health intervention programs (9).

As defined by the AVMA, therapeutic use of antimicrobials includes applications for prevention, control and treatment of disease (6). In the context of BRD, AMD are commonly used in feedlot cattle for BRD control (i.e., metaphylaxis) in certain groups of cattle (based on their BRD risk classification) in which there are already individuals with evidence of infectious disease and for individual treatment of clinically affected animals (6, 11). The antimicrobials used in risk group protocols for on-arrival use are specifically chosen based on the level of expected exposure to infectious agents, the types of pathogens most commonly encountered (past or present), the predicted ability of the host to mount an appropriate immune response, and in some cases past research documenting efficacy of the AMD in the different cattle populations. Appropriately applied BRD metaphylaxis has been shown to dramatically reduce the deleterious effects of BRD, improving animal health (13–15) and preventing significant economic loss to producers (16).

Liver abscesses are another important health and production problem in beef cattle, with a prevalence of between 10 and 20% in most feedlots (17). Liver abscesses vary in grading from mild to severe; all liver abscesses affect animal performance to some degree with the most severe abscesses reducing the value of beef carcasses by $38 per animal (18, 19). Acidotic conditions in the rumen lead to rumenitis, allowing the establishment of bacterial infections in the ruminal wall and the subsequent translocation of pyogenic bacteria, especially *Fusobacterium necrophorum* and *Trueperella pyogenes*, via the portal circulation to the liver. Ruminal acidosis is typically associated with sudden dietary changes to high energy diets, changes in feeding patterns, overly long intervals between feedings, and feeding of too little roughage. Inclusion of tylosin in feed, the most effective of the approved antimicrobials for liver abscess reduction incidence, has been shown by several studies to reduce liver abscesses in cattle in conventional feeding systems by 40–70% (20).

Data about AMU are collected for a variety of reasons, including the monitoring of usage trends over time, comparison of usage between different species or countries, benchmarking between hospitals, clinics, or farms, and studying the association between AMU and AMR (21). Five categories of requirements regarding measurement of AMU have been identified: level of resolution, comprehensiveness, stability of the measure over time, ability to assess exposure to AMD, and comparability of the measure between different populations. Various indicators of AMU are available and published; there can be wide discrepancies between the results obtained from different indicators applied to identical data, and no indicator fully meets all of the requirements for measurement of AMU. Selection of the appropriate indicator requires consideration of the study objectives and determination of which indicator best meets the needs of the study (21). To fully understand the role AMU plays in the selection of AMR in feedlot cattle, and to measure the potential effect of interventions, accurate AMU data must be available (22). Often, AMD sales data have been used as a proxy for AMD administration (23, 24). It is important, however, to recognize that sales data are not equivalent to use data and may result in use overestimation, because producers may not administer what is purchased. Furthermore, it is not always possible to correctly attribute the species in which a product is used because AMD are often authorized for use in multiple species. Detailed farm-level AMU data collection is therefore considered invaluable due to its more closely targeted nature, ensuring accurate assignation of species exposed to the AMD, ability to evaluate the indication of use and risk characteristics of exposed animals, and potential exploration of relationships of AMU with AMR in a meaningful way (25). Since AMU data collected for monitoring and surveillance are intended to address questions requiring detailed levels of information (26, 27) development of practical methods for on-farm collection of data should be prioritized.

In this study, a census sample of >2.6 million cattle entering feedlots in western Canada over ~4 years was performed to summarize AMU in this sector, increase knowledge of the indications for which AMD are administered, and evaluate trends in AMU over time. In addition to providing an unprecedented representation of AMU in fed cattle in western Canada, this study also sought to evaluate the development of methods for feedlot AMU monitoring.

## Materials and Methods

### Project Summary

Detailed data about AMD administered to cattle in 36 western Canadian feedlots from November 1, 2008 to October 31, 2012, including information about the specific AMD, dose administered, use indication, and exposed animal characteristics/demographics were collected. The AMU data were converted to indicators of frequency or numbers of animal daily doses (nADD) (28) or grams AMD (gAMD) per 100,000 cattle. Data were summarized and statistical analyses performed to determine relative risks of exposure to AMD or confidence intervals for binomial proportions where appropriate.

### Feedlots and Animals

Mixed-breed cattle placed in 36 western Canadian feedlots and fed for beef production from November 1, 2008 to October 31, 2012 (*n* = 2,615,082) were enrolled in the study. The cattle were divided into 4 placement cohorts (PC1, PC2, PC3, and PC4) based on date of arrival into the feedlot. Placement cohort 1 (PC1) included cattle arriving between November 1, 2008 and October 31, 2009, PC2 included cattle arriving between November 1, 2009 and October 31, 2010, PC3 included cattle arriving between November 1, 2010 and October 31, 2011, and PC4 included cattle arriving between November 1, 2011 and October 31, 2012. Cattle were owned and managed by multiple individuals and companies, but their healthcare was overseen by a single veterinary practice (Feedlot Health Management Services Ltd; Feedlot Health), who worked with feedlots to develop risk assessment algorithms and risk-based animal health and treatment protocols. This study population represented ~21.5% of fed cattle in Canada for the time period. The one-time capacity of 8 of the feedlots was <5,000 cattle, 5,000–9,999 for 15 of the feedlots, 10,000–20,000 for 5 of the feedlots, and >20,000 cattle for 8 of the feedlots.

The basic design of the feedlots and management strategies were representative of typical operations in western Canada; animals were housed in open air, dirt-floor pens arranged side by side with central feed alleys. Designated animal handling facilities with a hydraulic chute, individual animal scale, and chute-side computer for data recording were located at each site. Standardized animal health and treatment protocols developed by Feedlot Health directed parenteral and oral bolus AMD exposures of cattle, and prescriptions for in-feed AMD were written for feedlots by Feedlot Health veterinarians with valid veterinarian-client-patient relationships.

### Data Collection and Management

Using proprietary data collection and management software (*i*FHM*S*, Feedlot Health, Okotoks, AB), individual animal data were collected at initial processing and subsequent handling times. Individual animal identification included both a Canadian Cattle Identification Agency approved electronic tag (national ID) and a color-coded, uniquely numbered dangle ear tag (visual ID), with both tags recorded and correlated to the individual animal in the database. Data were compiled for analysis using Microsoft® Access 2010 (Microsoft Corporation, Redmond, WA) and Microsoft® Excel 2010. Information collected at arrival included date, unique animal identification number, sex, age category (calf or yearling), feedlot number, and production lot number. A production lot was defined as a group of cattle purchased together with similar characteristics. Risk assessment for BRD and assignment of status (high or low risk for development of BRD) was automatically performed by *i*FHM*S* at time of placement based on historical data and customized, if necessary, by veterinarians and other personnel working under their supervision. Information collected included unique identification number, date, animal weight at time of administration, active AMD ingredient, dosage, route, reason for administration (metaphylactic or treatment), and disease/syndrome [acute interstitial pneumonia/diphtheria, undifferentiated fever/BRD, lameness (arthritis, footrot, foot lesions), metabolic disease (bloat, grain overload, laminitis), nervous disease, eye disease, and other].

Data regarding in-feed AMU were assembled using a combination of approaches including Feedlot Health veterinarian feed AMD prescriptions, daily feed delivery data previously collected by Feedlot Health and stored in a database for consulting purposes, extraction of feed data from the feedlot's on-site computer system, obtaining a hard copy of the feed delivery records, or through combinations of these approaches. Data including unique production lot/feedlot combinations, feed delivery date, number of animals in the production lot each day, and number of animals receiving each type of in-feed AMD were compiled into Microsoft® Excel 2010. In-feed AMU was either reported as number of animals receiving a certain inclusion rate of AMD in feed (e.g., 35 mg chlortetracycline per kg dry matter) or the number of animals receiving a certain number of grams per head (e.g., 1 gram of chlortetracycline per head per day). Although cattle within a production lot could arrive over a span of days, they were typically assigned to the same placement cohort if the group arrived between 1 November and 31 October of the following year. However, if the production lot happened to arrive at the juncture of placement cohorts (i.e., was placed between 30 October and 2 November of the same year), the production lot was divided between the cohorts. In these cases, feed data were prorated to the appropriate placement cohort based on the percentage of animals in the production lot assigned to each placement cohort. Feed data were prorated according to risk category for BRD (high or low) according to the assessed BRD risk status of the animal at entry into the feedlot.

Data were summarized and metrics/indicators calculated using SAS® software (Windows version 9.4, SAS Institute, Cary, NC). For this study, AMD classified in categories I to III, or medium to very high importance to human medicine (8), are summarized and presented separately from category IV AMD of low importance to human medicine (e.g., ionophores).

### Metrics and Indicators

Metrics summarized included total gAMD and number of nADD for both individually dosed AMD (parenterally or orally administered) and AMD administered in feed to entire housing groups. Frequency of exposure (e.g., number of cattle exposed) was also summarized where appropriate.

For individually dosed AMD, total gAMD used were calculated by summing the administered mg of AMD recorded per animal. For AMD administered in feed at a given inclusion concentration, the mg of AMD per kg of feed was multiplied by the estimated average intake of daily feed per animal (29) to calculate the estimated daily intake of AMD per animal. This intake was then multiplied by the number of animals fed the ration daily to yield the amount of the particular AMD used. For AMD fed on a mg/animal basis, this dose was multiplied by the number of animals exposed to yield the daily amount of AMD used.

To calculate the number of standardized doses of individually administered AMD, an ADD_kg_ was assigned for each drug in mg/kg/day (Individually Dosed AMD Appendix, Supplementary Material). Use of tetracyclines, macrolides, fluoroquinolones, phenicols, cephalosporins, penicillin, sulfonamides, and potentiated sulfonamides was recorded. For long-acting AMD (effect lasting longer than 24 h), the administered mg/kg dosage was divided by the number of days of duration that a single dose of a particular product is assumed to maintain therapeutic concentrations in the target tissues to produce the ADD_kg_, based on pharmacokinetics and pharmacodynamics studies and the product label. An individual animal weight was recorded for ~95% of parenteral or oral bolus treatment, and a group average weight was recorded for 4.7% of these exposures. No weight was recorded for the remainder of exposures; in these instances, the mean weight at exposure was calculated for the type of AMD and this weight substituted. The calculation of the number of animal daily doses (nADD) of individually dosed AMD was performed for each administration via SAS software using the following equation:

$$\begin{array}{l} nADD=\frac{Qty\;of\;active\;substance\;in\;mg\;administered}{ADD\;{\left(mg\;per\;kg\;per\;day\right)}^{*}weight\;\left(kg\right)\;of\;animal} \end{array}$$

Calculations of the standardized doses for in-feed AMD were performed differently since the AMD were mixed in feed to target either mg of drug/kg dry matter of feed consumed or mg of drug administered per animal per day, and the number of cattle consuming the ration daily was recorded. Use of tetracyclines, tylosin, and ionophores was recorded in feed. For AMD where there was not a range of dosages administered across feedlots, 1 ADD was equal to 1 animal recorded as exposed to the in-feed AMD. In instances with a range of dosages, relative nADD were calculated by normalizing to the highest dose used. In instances with an inclusion rate based on mg per kg dry matter consumed, nADD were standardized based on feed intake estimates (In Feed AMD Appendix, Supplementary Material). For in-feed administration of chlortetracycline, which had the widest range of dosages reported for various indications, the average of the reported dosage range for metaphylaxis or treatment of *Histophilus somni* was used as the reference ADD, instead of the highest dose. For in-feed tylosin exposures, the in-feed dosage was normalized by the parenteral dosage labeled for use in respiratory disease in cattle.

Treatment frequency with an AMD was calculated as nADD/100,000 cattle. When the total number of animals in the population is the denominator, treatment frequency indicates how many days on average an animal in the population is treated with an AMD during the time of data collection (30). With these data, if the number of animals in the population was used as the denominator, most of the indicators would have been <0 and tables would have been difficult to read. Therefore, for convenience and easier reading, the denominator was multiplied by 100,000. Thus, treatment frequency in this context represents how many days on average 100,000 animals were treated with an AMD during the feeding period. Where suitable, the gAMD/100,000 cattle indicator was also calculated for comparison.

### Statistics

Relative risks of exposure to AMD were estimated using Poisson regression (Proc GENMOD, SAS v9.4, SAS Inc., Cary, NC) as previously described (31) using numbers of AMD exposures as the dependent variable and the assessed risk category of the animal for BRD as the independent variable. Robust error variances were estimated using the repeated statement and the individual identification number of the animal as the subject identifier. For estimation of percentages of cattle exposed to AMD for different reasons (**Table 7**), width-adjusted 95% confidence intervals (95% CI) for binomial proportions were calculated, adding 2 successes and 2 failures to actual counts as previously described (32).

## Results

### Demographics of the Cattle

Approximately 2.6 million cattle entered the 36 feedlot study sites over the 4-year period of the study and were followed until feedlot exit (Table 1). While the number of animals in each placement cohort slightly decreased over the study period, the placement numbers were fairly consistent from year to year. Overall, more males (63%) than females and more yearlings (55%) than calves were included in this study population, and the majority of animals entered the feedlot in the fall or winter (62%) and were classified as low risk (LR) for developing BRD (61%). The BRD risk category of the cattle placed over time was consistent [~39% categorized as high risk for developing BRD (HR)]. The cattle in these 36 feedlots in these 4 placement cohorts comprised 21.5% of the cattle fed during the same time period in Canada. Cattle assessed to be at HR for developing BRD tended to be calves that entered the feedlot in the fall or winter (Table 2). Sex of cattle did not appear to significantly influence risk categorization for BRD.

**Table 1:** Characteristics of cattle by placement cohort^a^, cattle placed 2008–2012.

	**Placement cohort**				
	**1**	**2**	**3**	**4**	**Total**
	***n* = 717,176**	***n* = 670,066**	***n* = 648,916**	***n* = 578,924**	***n* = 2,615,082**
**Characteristics**					
**AGE AT ARRIVAL, NO. (%)**					
Calf	333,742 (47)	314,190 (47)	288,484 (44)	244,083 (42)	1,180,499 (45)
Yearling	383,434 (53)	355,876 (53)	360,432 (56)	334,841 (58)	1,434,583 (55)
**SEX, NO. (%)**					
Male	453,222 (63)	399,396 (60)	420,739 (65)	370,171 (64)	1,643,528 (63)
Female	263,954 (37)	270,670 (40)	228,177 (35)	208,753 (36)	971,554 (37)
**SEASON OF ARRIVAL, NO. (%)**					
Fall or Winter	424,138 (59)	413,518 (62)	412,678 (64)	366,352 (63)	1,616,686 (62)
Spring or Summer	293,038 (41)	256,548 (38)	236,238 (36)	212,572 (37)	998,396 (38)
**BRD**^**b**^ **RISK CATEGORY, NO. (%)**					
High	269,404 (38)	265,033 (40)	259,667 (40)	227,535 (39)	1,021,639 (39)
Low	447,772 (62)	405,033 (60)	389,249 (60)	351,389 (61)	1,593,443 (61)

*The number of cattle are presented in the left part of the cell with the % of the total number of cattle in the placement cohort this number represents in the right part of the cell, in parentheses. Percentages of use may not add to 100% due to rounding*.

a *Placement cohort comprised of cattle placed in the feedlot between 1 November and 31 October of consecutive years*.

b *Bovine Respiratory Disease*.

**Table 2:** Characteristics of cattle overall and stratified by risk for bovine respiratory disease (BRD) assessed at placement, cattle placed 2008–2012.

	**Overall**	**High risk** **for BRD**	**Low risk** **for BRD**
	***n* = 2,615,082**	***n* = 1,021,639**	***n* = 1,593,443**
**Characteristics**			
**AGE AT ARRIVAL, NO. (% OF TOTAL, % OF BRD RISK GROUP)**			
Calf	1,180,499 (45)	950,197 (36, 93)	230,302 (9, 14)
Yearling	1,434,583 (55)	71,442 (3, 7)	1,363,141 (52, 86)
**SEX, NO. (% OF TOTAL, % OF BRD RISK GROUP)**			
Male	1,643,528 (63)	662,896 (25, 65)	980,632 (37, 62)
Female	971,554 (37)	358,743 (14, 35)	612,811 (23, 38)
**SEASON OF ARRIVAL, NO. (% OF TOTAL, % OF BRD RISK GROUP)**			
Fall or Winter	1,616,686 (62)	836,927 (32, 82)	779,759 (30, 49)
Spring or Summer	998,396 (38)	184,712 (7, 18)	813,684 (31, 51)

*The number of cattle with the characteristic is presented in the left part of the cell. For the Overall column, the % of cattle that this number represents is presented in the right part of the cell in parentheses. For the High Risk for BRD and Low Risk for BRD columns, in parentheses and separated by a comma, first the % of total cattle is presented, then the % of cattle in that risk category is presented. Percentages of use may not add to 100% due to rounding*.

### Overall AMU

**Figure 1:**
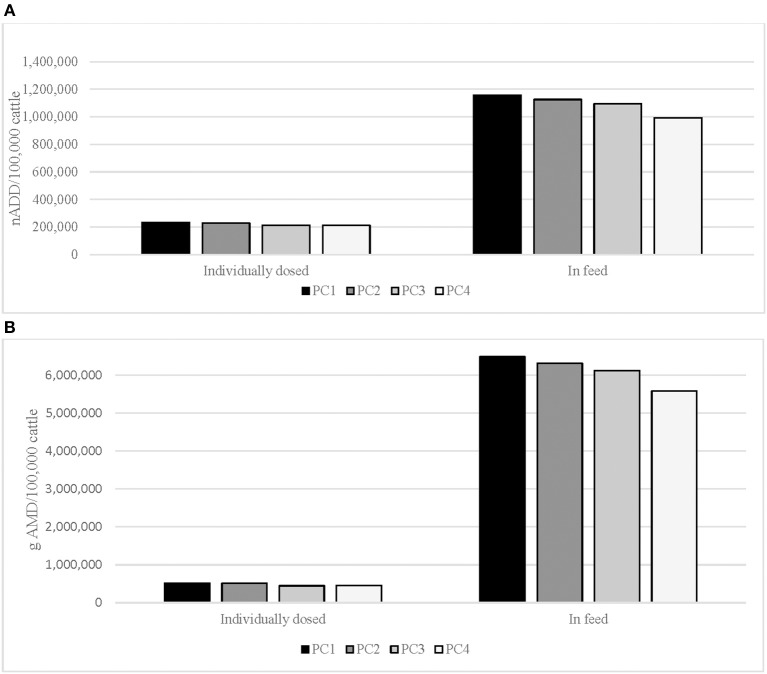
**(A)** Individually dosed and in-feed antimicrobial drug use (AMU) in number of animal daily doses (nADD)/100,000 cattle in placement cohort^a^ (PC), cattle placed 2008–2012. **(B)** Individually dosed and in-feed antimicrobial drug use in total grams of antimicrobial drug (gAMD)/100,000 cattle in placement cohort^a^ (PC), cattle placed 2008–2012. ^a^Placement cohort comprised of cattle placed in the feedlot between 1 November and 31 October of consecutive years.

Substantially more medically important AMD were used in-feed than dosed individually, whether measured with the nADD/100,000 cattle or total gAMD/100,000 cattle metric (Table 3, Figures 1A,B). When calculated with nADD, 5 times as much medically important AMD were used in-feed than was administered to individual cattle through the study period; when gAMD were used as the metric, almost 13 times as much AMD was used in-feed. A reduction in individually dosed (average 11%) and in-feed medically important AMU (average 14%) over the study period was evident using both indicators (Figures 1A,B).

**Table 3:** Individually dosed and in-feed antimicrobial drug use (AMU) in number of animal daily doses (nADD) and total grams of antimicrobial drug (gAMD) by placement cohort^a^, cattle placed 2008–2012.

		**Placement cohort**				
		**1**	**2**	**3**	**4**	**Total**
		**(*n* = 717,176)**	**(*n* = 670,066)**	**(*n* = 648,916)**	**(*n* = 578,924)**	**(*n* = 2,615,082)**
**nADD or gAMD, NO. (NO./100,000 CATTLE)**						
Individual	nADD	1,680,387 (234,306)	1,532,732 (228,743)	1,383,193 (213,154)	1,226,748 (211,901)	5,823,060 (222,672)
	gAMD	3,674,494 (512,356)	3,462,134 (516,685)	2,899,241 (446,782)	2,611,049 (451,018)	12,646,918 (483,615)
In Feed	nADD	8,300,631 (1,157,405)	7,539,570 (1,125,198)	7,105,901 (1,095,042)	5,744,496 (992,271)	28,690,598 (1,097,120)
	gAMD	46,488,463 (6,482,155)	42,304,541 (6,313,489)	39,735,901 (6,123,428)	32,315,393 (5,581,975)	160,844,298 (6,150,641)

*The total nADD or gAMD is presented on the left side of the cell, and the nADD/100,000 cattle or gAMD/100,000 cattle is presented on the right side of the cell in parentheses*.

a *Placement cohort comprised of cattle placed in the feedlot between 1 November and 31 October of consecutive years*.

### AMU by Drug Class and Type of AMD

**Figure 2:**
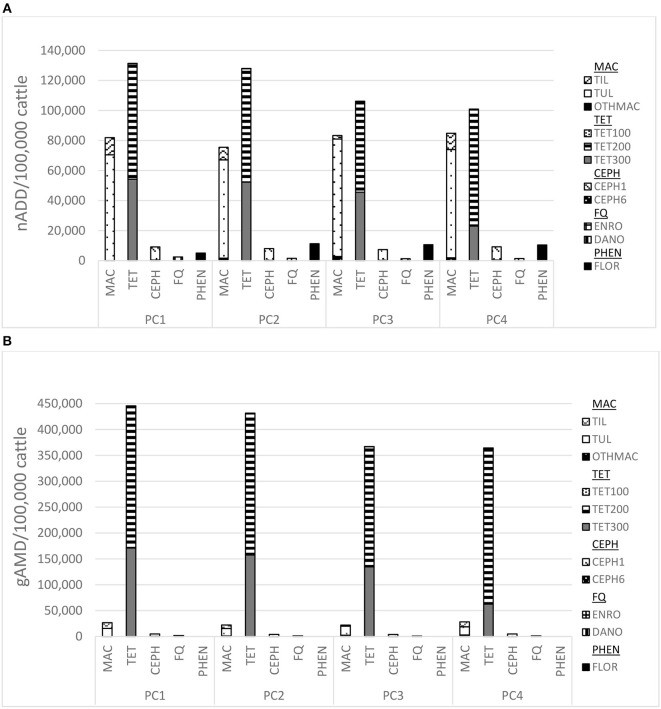
**(A)** Individually dosed antimicrobial drug use in nADD/100,000 cattle by placement cohort (PC)^a^, antimicrobial class^b^, and specific type of antimicrobial drug^c^, cattle placed 2008–2012. **(B)** Individually dosed antimicrobial drug (AMD) use in grams AMD (gAMD)/100,000 cattle by placement cohort (PC)^a^, antimicrobial class^b^, and specific type of antimicrobial drug^c^, cattle placed 2008–2012. ^a^Placement cohort comprised of cattle placed in the feedlot between 1 November and 31 October of consecutive years. ^b^MAC, macrolides; TET, tetracyclines; CEPH, third generation cephalosporins; FQ, fluoroquinolones; PHEN, phenicols (TMS, trimethoprim-sulfamethoxazole; PEN, penicillin; SULF, sulfonamides not depicted due to low usage; ^c^TIL, tilmicosin 10 mg/kg; TUL, tulathromycin 2.5 mg/kg; OTHMAC, gamithromycin 6 mg/kg, tildipirosin 4 mg/kg, tylosin 29 mg/head; TET100, oxytetracycline 6.67 mg/kg; TET200, oxytetracycline 20 mg/kg; TET300, oxytetracycline 30 mg/kg; CEF1, ceftiofur hydrochloride or sodium, 1.1 mg/kg; CEF6, ceftiofur crystalline free acid 6.6 mg/kg; DANO, danofloxacin 6 mg/kg; ENRO, enrofloxacin 7.7 mg/kg; FLOR, florfenicol 40 mg/kg.

Employing the nADD/100,000 cattle indicator (Figure 2A), tetracyclines were the class of AMD most commonly administered to individual cattle (52.8% of total individually dosed usage). Macrolides were the second most common AMD class administered to individual cattle (36.5%), with tulathromycin constituting the majority of this antimicrobial class use (88.0%). While tetracycline use decreased over time by 23.1%, macrolide use slightly increased over the course of the study (3.6%). Macrolide use appeared markedly lower when assessed using the gAMD/100,000 cattle metric (Figure 2B); based on this indicator tetracycline use comprised 83.5% of individually dosed AMU while macrolides represented only 5%.

**Figure 3:**
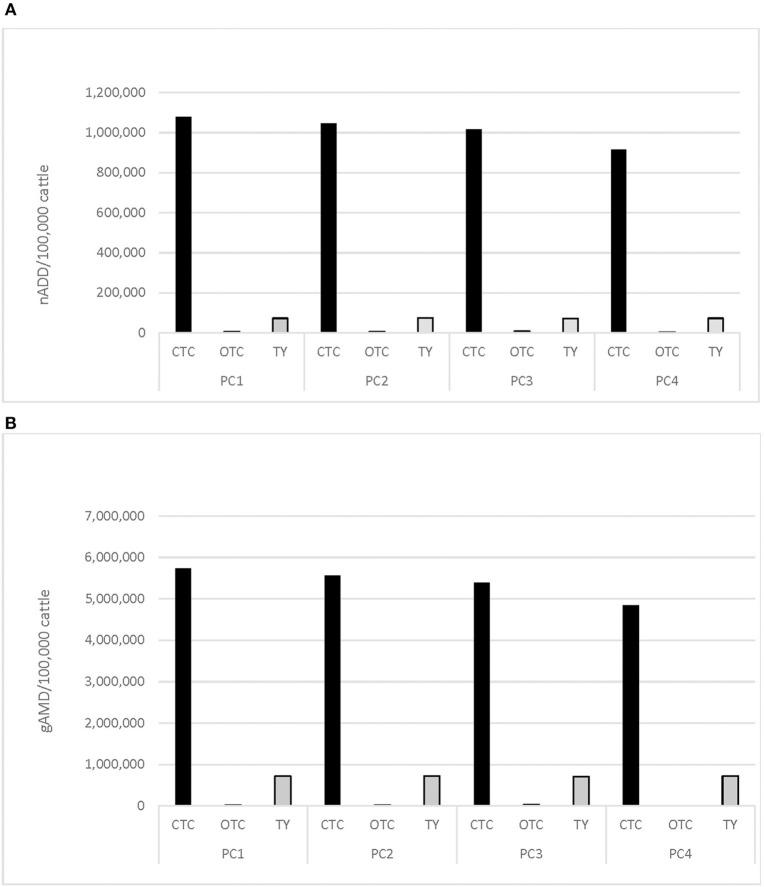
**(A)** In-feed antimicrobial drug use in nADD/100,000 cattle by placement cohort (PC)^a^, and antimicrobial class^b^, cattle placed 2008–2012. **(B)** In-feed antimicrobial drug use in grams AMD (gAMD)/100,000 cattle by placement cohort (PC)^a^, and antimicrobial class^b^, cattle placed 2008–2012. ^a^Placement cohort comprised of cattle placed in the feedlot between 1 November and 31 October of consecutive years. ^b^CTC, chlortetracycline; OTC, oxytetracycline; TY, tylosin.

Employing either the nADD/100,000 cattle or gAMD/100,000 cattle metric, the majority of medically important AMD administered in-feed was tetracycline (Figures 3A,B), followed by tylosin. When the nADD indicator was employed, there was 14 times as much tetracycline (chlortetracycline and oxytetracycline) used as tylosin; when the gAMD indicator was employed, there was 7.5 times as much tetracycline used as tylosin over the course of the study.

### AMU by Indication, Risk Class for BRD, and Cohort

Overall, using nADDs, category III AMD comprised the greatest amount of individually dosed AMU (56.9%); 38.5% of individually dosed AMU were category II AMD and 4.6% of individually dosed AMU were category I AMD (Table 4). The bulk of individually dosed AMU (92.9%) was administered to prevent or treat BRD occurrence, with the majority of this use (89.9%) administered as BRD metaphylaxis. Of the category I AMD use, 83.2% was comprised of third-generation cephalosporins; 44.3% was administered to treat infectious causes of lameness and 24.3% was used to treat other miscellaneous infectious diseases (ocular diseases, infectious neurologic disorders, etc.).

**Table 4:** Individually dosed antimicrobial use by indication^a^^,^^b^ and class of antimicrobial drug (AMD)^c^, organized by category of importance to human medicine^d^ and expressed in number of animal daily doses (nADD).

		**Indication**						
		**ARD**	**UF/BRD**		**Lameness**	**Other**	**Implant**	**Total**
	**AMD** **Class**		**Metaphylaxis**	**Treatment**				
		**nADD No. (% of use of specific AMD class, % of use for specified indication)**						
I	FQ	3 (0.01, 0.01)	0 (0, 0)	44,192 (99.4, 7.9)	71 (0.2, 0.03)	181 (0.4, 0.1)	0 (0, 0)	44,447
	CEPH	33,075 (15.0, 98.7)	0 (0, 0)	51,498 (23.3, 9.2)	99,515 (45.1, 44.3)	36,604 (16.6, 24.3)	0 (0, 0)	220,692
II	MAC	85 (0.004, 0.25)	1,975,173 (93.0, 40.7)	143,019 (6.7, 25.6)	602 (0.03, 0.3)	2,579 (0.1, 1.7)	3,061 (0.1, 100)	2,124,519
	PEN	3 (0.2, 0.01)	0 (0, 0)	13 (0.8, 0.002)	1,347 (81.7, 0.6)	286 (17.3, 0.2)	0 (0, 0)	1,649
	TMS	214 (0.2, 0.6)	0 (0, 0)	659 (0.6, 0.1)	72,733 (62.8, 32.4)	42,146 (36.4, 28.0)	0 (0, 0)	115,752
III	TET	64 (0.002, 0.2)	2,876,561 (93.6, 59.3)	81,634 (2.7, 14.6)	49,860 (1.6, 22.2)	64,933 (2.1, 43.2)	0 (0, 0)	3,073,052
	PHEN	74 (0.03, 0.2)	584 (0.2, 0.01)	238,256 (98.6, 42.6)	360 (0.1, 0.2)	2,258 (0.9, 1.5)	0 (0, 0)	241,532
	SULF	0 (0, 0)	0 (0, 0)	20 (1.4, 0.003)	8 (0.6, 0.004)	1,389 (98.0, 0.9)	0 (0, 0)	1,417
	ALL	33,518	4,852,318	559,291	224,496	150,376	3,061	5,823,060

*The nADD is presented on the left side of the cell. In parentheses on the right side of the cell, the % of use of the specific AMD class is first presented, then the % of use for specified indication presented separated by a comma. Percentages of use may not add to 100% due to rounding. Darkening green color indicates increasing nADD*.

a ARD, acute respiratory disease; UF/BRD, undifferentiated fever/bovine respiratory disease; Other, neurologic, metabolic, ocular, or other indications; Implant, antimicrobial associated with implantation of hormone.

b Metaphylaxis, group administration of an antimicrobial to a population at risk for disease before overt clinical disease is apparent in the entire group; Treatment, administration of an antimicrobial to an individual animal diagnosed with clinical disease.

c FQ, fluoroquinolones; CEPH, 3rd generation cephalosporins; MAC, macrolides; PEN, penicillins; TMS, trimethoprim-sulfamethoxazole; TET, tetracyclines; PHEN, phenicols; SULF, sulfonamides.

d Roman numerals I to III signify categories of importance to human medicine as designated by the Canadian Veterinary Drugs Directorate.

Most individually dosed AMD administered for BRD metaphylaxis were tetracycline class drugs (59.3%), followed by macrolides (40.7%) and a small amount of phenicol class drugs (Table 5). Cattle assessed to be at HR for developing BRD were about 1.6 times more likely to receive metaphylaxis for BRD than LR cattle, and about 4 times as likely to be treated for BRD (Tables 5, 6). High risk cattle were over 100 times more likely to receive a macrolide for BRD metaphylaxis than cattle assessed to be LR. Conversely, HR cattle were about a third less likely to receive tetracycline for BRD metaphylaxis than LR cattle. Of the AMD used for treatment of BRD, phenicols comprised the highest percentage (42.6%); HR cattle were 2.3 times more likely to receive a phenicol for this purpose than LR cattle. Other AMD used for treatment of BRD, listed in descending amount of usage, were macrolides (25.6%), tetracyclines (14.6%), cephalosporins (9.2%), fluoroquinolones (7.9%), potentiated sulfonamides, sulfonamides, and penicillin (all <0.01).

**Table 5:** Individually administered antimicrobial use by antimicrobial class^a^ for bovine respiratory disease (BRD) in total number of animal daily doses (nADD), stratified by reason for exposure, and risk category for BRD with relative risk of antimicrobial exposure for cattle assessed to be high risk (HR) or low risk (LR) for BRD.

		**Reason for Exposure**		**Assessed risk category for BRD**	
		**Metaphylaxis^**b**^**	**Treatment^**c**^**	**HR**	**LR**
	**AMD Class**	**nADD** **(% of total for column)**			
I	FQ	0	44,192	34,713	9,479
		(0)	(7.9)	(1.0)	(0.5)
	CEPH	0	51,498	22,911	28,587
		(0)	(9.2)	(0.7)	(1.4)
II	MAC	1,975,173	143,019	2,049,808	68,384
		(40.7)	(25.6)	(60.9)	(3.3)
	PEN	0	13	13	0
		(0)	(<0.01)	(<0.01)	(0)
	TMS	0	659	131	528
		(0)	(<0.01)	(<0.01)	(<0.01)
III	TET	2,876,561	81,634	1,060,891	1,897,304
		(59.3)	(14.6)	(31.5)	(92.8)
	PHEN	584	238,256	198,043	40,797
		(0)	(42.6)	(5.9)	(2.0)
	SULF	0	20	16	4
		(0)	(<0.01)	(<0.01)	(<0.01)
	ALL	4,852,318	559,291	3,366,526	2,045,083
		(100)	(100)	(100)	(100)

*The nADD is presented in the top of the cell, and the % of the total nADD by reason or assessed risk category for BRD that this number represents is presented in parentheses in the bottom of the cell. Percentages of use may not add to 100% due to rounding*.

a *FQ, fluoroquinolones; CEPH, 3rd generation cephalosporins; MAC, macrolides; PEN, penicillin; TMS, trimethoprim-sulfamethoxazole; TET, tetracyclines; PHEN, phenicols; SULF, sulfonamides. Roman numerals I to III signify category of importance to human medicine as designated by the Canadian Veterinary Drugs Directorate*.

b *Metaphylaxis is the group administration of an antimicrobial to a population at risk for disease before overt clinical disease is apparent in the entire group*.

c *Treatment is the administration of an antimicrobial to an individual animal diagnosed with clinical disease*.

**Table 6:** Output of univariate regression analysis for estimates of relative risk of exposure to specific antimicrobial drugs (AMD) for indications of metaphylaxis^a^ or treatment^b^ of bovine respiratory disease (BRD) in cattle classified as high risk (HR) for developing BRD and cattle classified as low risk (LR) for developing BRD.

**AMD**	**Indication**	**Regression coefficient**	***p* value**	**Relative risk estimate**	**95% confidence interval**
Any	BRD (Metaphylaxis)	0.49	<0.0001	1.63	1.629–1.634
	BRD (Treatment)	1.40	<0.0001	4.07	4.025–4.114
Macrolide	BRD (Metaphylaxis)	4.62	<0.0001	101.55	98.976–104.199
	BRD (Treatment)	−0.16	<0.0001	0.85	0.838–0.867
Tetracycline	BRD (Metaphylaxis)	−1.04	<0.0001	0.35	0.352–0.354
	BRD (Treatment)	0.08	<0.0001	1.08	1.057–1.010
Phenicol	BRD (Metaphylaxis)	−0.26	0.07	0.77	0.583–1.023
	BRD (Treatment)	0.84	<0.0001	2.31	2.273–2.348

a *Metaphylaxis is the group administration of an antimicrobial to a population at risk for disease before overt clinical disease is apparent in the entire group*.

b *Treatment is the administration of an antimicrobial to an individual animal diagnosed with clinical disease*.

Most in-feed medically important AMU was related to prevention and treatment of liver abscesses (59.5%) and prevention and treatment of histophilosis (40.2%) (Table 7). Of in-feed medically important AMU, 92.8% was chlortetracycline (category III AMD), comprising 100% of use for histophilosis, 88.1% of use for liver abscesses, and about half of use for “other” indications. Macrolides (category II AMD) were only used for prevention and treatment of liver abscesses and made up 11% of AMU for this purpose.

**Table 7:** In-feed antimicrobial use by indication and class of medically important antimicrobial drug (AMD)^a^, organized by category of importance to human medicine^b^ and expressed in total number of animal daily doses (nADD).

			**Indication**			
			**Histophilosis**	**Liver abscesses**	**Other**	**Total**
**AMD Class**		**AMD**	**nADD No. (% of use of specific AMD class, % of use for specified indication)**			
II	MAC	Tylosin	0 (0, 0)	1,903,454 (100.0, 11.1)	0 (0, 0)	1,903,454
III	TET	Chlortetracycline	11,531,483 (43.3, 100.0)	15,052,190 (56.5, 88.1)	37,815 (0.1, 49.6)	26,621,488
		Oxytetracycline	0 (0, 0)	127,187 (76.8, 0.7)	38,468 (23.2, 50.4)	165,655
		Total	11,531,483	17,082,831	76,283	28,690,597

*The nADD are presented in the left part of the cell, and the % of the use of the specific AMD class and the % of use for the specified indication are presented in the right part of the cell separated by a comma. Percentages of use may not add to 100% due to rounding*.

a *MAC, macrolides; TET, tetracyclines*.

b *Roman numerals I to III designate categories of importance to human medicine as designated by the Canadian Veterinary Drugs Directorate*.

Overall, throughout the course of the study 97% of cattle were exposed to medically important AMD in feed, 73% were individually dosed with AMD, and 21% received tylosin as part of a hormonal growth implant. The percentage of cattle exposed to AMD in-feed or as part of growth implants did not appear to be influenced significantly by age, sex, season of arrival, or assessed risk category for BRD (range of exposure 95–98% for in-feed AMD and 19–24% for implants). Conversely, higher percentages of cattle that were calves, male, arriving in cold weather, and assessed to be at HR for developing BRD were exposed to individually dosed AMD (Table 8); 95% of cattle assessed to be at HR for developing BRD were exposed to individually dosed AMD compared to 59% of LR cattle.

**Table 8:** Number and percentage of cattle (placed 2008–2012) exposed to antimicrobial drugs in-feed, individually dosed, and associated with hormone implants.

		**Type of antimicrobial exposure**^**b**^		
	**All cattle**	**In feed**	**Individually dosed**	**With implant**
**OVERALL, NO. (% OF TOTAL CATTLE)**				
	2,615,082 (100)	2,527,316 (97)	1,910,825 (73)	544,790 (21)
**AGE AT ARRIVAL, NO. (% OF TOTAL CATTLE, % OF CALVES OR YEARLINGS)**				
Calf	1,180,499 (45)	1,151,277 (44, 97)	1,060,838 (41, 90)	254,633 (10, 22)
Yearling	1,434,583 (55)	1,376,039 (53, 96)	849,987 (33, 59)	290,157 (11, 20)
**SEX, NO. (% OF TOTAL CATTLE, % OF SPECIFIC ROUTE OF EXPOSURE)**				
Male	1,643,528 (63)	1,607,049 (61, 98)	1,291,246 (49, 79)	327,661 (13, 20)
Female	971,554 (37)	920,267 (35, 95)	619,579 (24, 64)	217,129 (8, 22)
**SEASON OF ARRIVAL, NO. (% OF TOTAL CATTLE, % OF SPECIFIC ROUTE OF EXPOSURE)**				
Cold	1,616,686 (62)	1,565,713 (60, 97)	1,314,050 (50, 81)	357,547 (14, 22)
Warm	998,396 (38)	961,603 (37, 96)	596,775 (23, 60)	187,243 (7, 19)
**BRD**^**a**^ **RISK CATEGORY, NO. (% OF TOTAL CATTLE, % OF SPECIFIC ROUTE OF EXPOSURE)**				
High	1,021,639 (39)	1,005,810 (38, 98)	971,146 (37, 95)	248,377 (9, 24)
Low	1,593,443 (61)	1,521,506 (58, 95)	939,679 (36, 59)	296,413 (11, 19)

*The number of exposed cattle is presented in the left part of the cell*.

*For the overall number, the percentage of overall cattle this number represents is presented in the right part of the cell in parentheses. When cattle are stratified by particular characteristics and type of antimicrobial exposure, first the percentage of total cattle is presented in parentheses, followed by the percentage of cattle with that characteristic. Percentages of use may not add to 100% due to rounding*.

a *Bovine Respiratory Disease*.

b *Individual cattle may be exposed to antimicrobial drugs via more than one route*.

Over 1.8 million cattle were exposed individually for metaphylaxis or treatment of BRD; 70.1% of cattle overall received individually dosed metaphylaxis for BRD and 5.9% were treated individually for BRD (Table 9). The percentage of cattle receiving individually dosed BRD for metaphylaxis and treatment both decreased slightly over the course of the study (2.9 and 1.3%, respectively). The percentage of cattle receiving AMD treatment for reasons other than BRD increased slightly (0.3%).

**Table 9:** Number and percentage, with 95% confidence interval (CI), of cattle (placed 2008-2012) exposed to individually dosed AMD for different indications by placement cohort (PC)^a^.

	**Placement cohort**				
	**1**	**2**	**3**	**4**	**Total**
	**(*n* = 717,176)**	**(*n* = 670,066)**	**(*n* = 648,916)**	**(*n* = 578,924)**	**(*n* = 2,615,082)**
**CATTLE EXPOSED, NO. (%OF PC; 95% CI)**					
Metaphylaxis^b^ or Treatment^c^ for BRD^d^	528,117 (73.6; 73.54–73.74)	492,271 (73.5; 73.36–73.57)	452,987 (69.8; 69.70–69.92)	408,678 (70.6; 70.48–70. 71)	1,882,053 (72.0;71.91–72.02)
Metaphylaxis for BRD	513,897 (71.7; 71.55–71.76)	481,549 (71.9; 71.76–71.97)	440,938 (67.9; 67.84–68.06)	398,082 (68.8; 68.64–68.88)	1,834,466 (70.1;70.09–70.20)
Treatment for BRD	48,220 (6.7; 6.67–6.78)	39,278 (5.9; 5.81–5.92)	34,652 (5.3; 5.29–5.39)	31,099 (5.4; 5.31–5.43)	153,249 (5.9; 5.83–5.89)
Treatment for Reason other than BRD	33,359 (4.7; 4.60–4.70)	26,030 (3.9; 3.84–3.93)	24,261 (3.7; 3.69–3.78)	28,722 (5.0; 4.91–5.02)	112,372 (4.3; 4.27–4.32)

The number of exposed cattle is presented in the left side of the cell. On the right side of the cell, the estimate of the percentage of cattle in the placement cohort this number represents is presented with the 95% CI to the right after a semi-colon.

a Placement cohort comprised of cattle placed in the feedlot between l November and 31 October of consecutive years.

b Metaphylaxis is the group administration of an antimicrobial to a population at risk for disease before overt clinical disease is apparent in the entire group.

c Treatment is administration of an antimicrobial to an individual animal diagnosed with clinical disease.

d *Bovine Respiratory Disease*.

Considering the use of class I AMD over time (Table 10), the use of fluoroquinolones decreased from 2,442 ADD/100,000 cattle at risk to 1,448 ADD/100,000 cattle at risk (40.7%) while the use of cephalosporins decreased from PC1 to PC3 from 9,135 ADD/100,000 cattle at risk to 7,379 ADD/100,000 cattle at risk (19%), but then increased in PC4 back to the PC2 level. Class I AMD were all individually administered and comprised 0.8% of all medically important AMU (in-feed and individually dosed). For class II AMD, the use of individually dosed macrolides increased very slightly from PC1 to PC4 (3.2%) but use over time appeared to remain fairly consistent as the overall average use was similar to that used by PC1. Similarly, in-feed macrolide use remained consistent over the course of the study. The use of penicillin and potentiated sulfonamides was low and stable. When use of in-feed and individually dosed medically important AMD were summed, class II AMD use comprised 12% of all AMD use. Summed in-feed and individually dosed class III AMD use made up 87% of all medically important AMD use, with the majority of this being tetracyclines (99% overall and 90% of medically important in-feed use). Over time, the use of individually dosed tetracycline and in-feed tetracycline decreased significantly overall from 1,215,633 ADD/100,000 cattle at risk to 1,020,057 ADD/100,000 cattle at risk (16.1%; 23.1% individually dosed and 15.2% in feed). Individually dosed phenicol use doubled, while sulfonamide use was both light and decreased over time.

**Table 10:** Medically important antimicrobial use (all routes) by indication and class/type of antimicrobial drug (AMD)^a^, organized by category of importance to human medicine^b^, and expressed in number of animal daily doses (nADD).

		**Placement cohort (PC)**^**c**^				
		**1**	**2**	**3**	**4**	**Total**
**CATTLE AT RISK, NO**.						
		717,176	670,066	648,916	578,924	2,615,082
**CATTLE EXPOSED TO AMD PARENTERALLY, NO. (% OF CATTLE AT RISK IN PC)**						
		537,599 (75)	498,618 (74)	457,940 (71)	416,668 (72)	1,910,825 (73)
**CATTLE EXPOSED TO AMD IN FEED, NO. (% OF CATTLE AT RISK IN PC)**						
		694,890 (97)	655,100 (98)	624,899 (96)	552,427 (95)	2,527,316 (97)
**nadd, NO. (NO./100,000 CATTLE AT RISK)**						
I	FQ^id^	17,512 (2,442)	10,197 (1,522)	8,356 (1,288)	8,382 (1,448)	44,447 (1,700)
	CEPH^id^	65,512 (9,135)	53,934 (8,049)	47,881 (7,379)	53,366 (9,218)	220,693 (8,439)
II	MAC^id^	587,157 (81,871)	505,405 (75,426)	539,728 (83,174)	489,167 (84,496)	2,121,457 (81,124)
	MAC^if^	524,514 (73,136)	492,342 (73,477)	462,974 (71,346)	423,624 (73,174)	1,903,454 (72,788)
	PEN^id^	687 (96)	492 (73)	415 (64)	55 (9)	1,649 (63)
	TMS^id^	30,402 (4,239)	28,785 (4,296)	27,852 (4,292)	28,712 (4,960)	115,751 (4,426)
III	TET^id^	942,109 (131,364)	857,577 (127,984)	688,886 (106,160)	584,480 (100,960)	3,073,052 (117,513)
	TET^if^	7,776,118 (1,084,269)	7,047,227 (1,051,721)	6,642,928 (1,023,696)	5,320,871 (919,097)	26,787,144 (1,024,333)
	PHEN^id^	36,334 (5,066)	75,732 (11,302)	68,938 (10,624)	60,528 (10,455)	241,532 (9,236)
	SULF^id^	497 (69)	396 (59)	245 (38)	279 (48)	1,417 (54)
	TOTAL	9,980,842 (1,391,686)	9,072,087 (1,353,909)	8,488,203 (1,308,059)	6,969,464 (1,203,865)	34,510,596 (1,319,676)

*Where number of cattle are presented, the number of cattle is presented to the left the cell with the % of cattle this number represents in the placement cohort in parentheses to the right of the cell. Where nADD are presented, the nADD is presented in the left part of the cell and the nADD/100,000 cattle is presented in the right part of the cell in parentheses*.

a FQ, fluoroquinolones; CEPH, 3rd generation cephalosporins; MAC, macrolides; PEN, penicillin; TMS, trimethoprim-sulfamethoxazole; TET, tetracyclines; PHEN, phenicols; SULF, sulfonamides. The superscript “id” indicates individually dosed and the superscript “if” indicates in feed.

b Roman numerals I to III designate category of importance to human medicine as designated by the Canadian Veterinary Drugs Directorate.

c *Placement cohort comprised of cattle placed in the feedlot between 1 November and 31 October of consecutive years*.

Examining trends over time in specific in-feed AMU (Table 11), the use of tetracyclines for prevention and treatment of respiratory disease (histophilosis) decreased over time from 500,713 ADD/100,000 cattle at risk to 326,174 ADD/100,000 cattle at risk (34.9%). In feed AMU for liver abscess prevention was consistent over the course of the study for both tetracyclines and macrolides. The use of tetracyclines for other indications (i.e., pododermatitis and keratoconjunctivitis) comprised only a small amount of overall in-feed use (0.3%) and varied from cohort to cohort.

**Table 11:** In-feed antimicrobial use, by placement cohort^a^, antimicrobial class^b^, and indication expressed in number of animal daily doses (nADD) and nADD/100,000 cattle at risk, cattle placed 2008–2012.

	**Placement cohort**			
	**1**	**2**	**3**	**4**
**Cattle at risk**	***n* = 717,176**	***n* = 670,066**	***n* = 648,916**	***n* = 578,924**
**nADD, No. (No./100,000 cattle at risk)**				
**HISTOPHILOSIS THERAPY**				
CTC (1 g/head)	465,982 (64,975)	406,967 (60,735)	497,428 (76,655)	440,371 (76,067)
CTC (4–7 g/head)	3,125,013 (435,739)	2,826,681 (421,851)	2,321,111 (357,691)	1,447,931 (250,107)
Total	3,590,995 (500,713)	3,233,648 (482,586)	2,818,539 (434,346)	1,888,302 (326,174)
**LIVER ABSCESSES**				
CTC (35 mg/kg DM)	4,131,126 (576,027)	3,748,300 (559,393)	3,763,010 (579,892)	3,409,754 (588,981)
OTC (11 mg/kgDM)	40,071 (5,587)	31,987 (4,774)	39,754 (6,126)	15,375 (2,656)
TY (11 mg/kg DM)	524,514 (73,136)	492,342 (73,477)	462,974 (71,346)	423,624 (73,174)
Total	4,695,711 (654,750)	4,272,629 (637,643)	4,265,738 (657,364)	3,848,753 (664,811)
**OTHER USE (EXAMPLES: PODODERMATITIS, KERATOCONJUNCTIVITIS)**				
CTC (1 g/head)	5,044 (703)	21,643 (3,230)	6,938 (1,069)	4,191 (724)
OTC (1–3 g/head)	8,881 (1,238)	11,650 (1,739)	14,687 (2,263)	3,250 (561)
Total	13,925 (1,942)	33,293 (4,969)	21,625 (3,332)	7,441 (1,285)

*The nADD is presented to the left of the cell, with the nADD/100,000 presented to the right of the cell in parentheses*.

a *Placement cohort comprised of cattle placed in the feedlot between 1 November and 31 October of consecutive years*.

b *CTC, chlortetracycline; OTC, oxytetracycline; TY, tylosin; DM, dry matter*.

### Ionophore Use

The ionophores monensin and lasalocid were used during the study. Using nADD, the use of ionophores comprised >89% of in-feed AMU overall (medically and non-medically important). Monensin was the most widely fed ionophore, constituting 99.9% of total use (Table 12), and use was consistent over time from cohort to cohort.

**Table 12:** In-feed ionophore use, by placement cohort^a^ and ionophore type^b^, expressed in number of animal daily doses (nADD), cattle placed 2008–2012.

	**Placement cohort**				
	**1**	**2**	**3**	**4**	**Total**
**Cattle at risk**	***n* = 717,176**	***n* = 670,066**	***n* = 648,916**	***n* = 578,924**	***n* = 2,615,082**
**nADD, NO. (NO./100,000 CATTLE AT RISK)**					
MON	69,129,832 (9,639,173)	64,538,569 (9,631,673)	63,584,520 (9,798,575)	57,007,350 (9,847,122)	254,260,271 (9,722,841)
LAS	0	0	0	92,337 (15,950)	92,337 (3,531)
ALL	69,129,832 (9,639,173)	64,538,569 (9,631,673)	63,584,520 (9,798,575)	57,099,687 (9,863,071)	254,352,608 (9,726,372)

*The nADD is presented at the top of the cell with the nADD/100,000 cattle presented at the bottom of the cell in parentheses*.

a *Placement cohort comprised of cattle placed in the feedlot between 1 November and 31 October of consecutive years*.

b *MON, monensin; LAS, lasalocid; ALL, all ionophores*.

## Discussion

The comprehensiveness and scope of this study provide an unprecedented representation of AMU in the Canadian feedlot sector in a large population of cattle managed by the same veterinary practice. While these data would ideally encompass a more recent period for the most timely estimates and descriptions of use, they nevertheless provide a baseline and practical information about methodological approaches. The thorough data collection allowed for not only an examination of general AMU trends, but also detailed evaluation of reasons for use and specific characteristics of exposed cattle.

Overall, if all AMD categories were considered together, the use of category IV (non-medically important) ionophores in feed comprised the majority of AMU in this population of beef cattle on an nADD basis. This fact underscores the importance of transparency in reporting AMD categories in AMU. These data demonstrate the huge potential for variability in the summary measures for AMU in beef cattle, depending upon inclusion or exclusion of ionophores. In this dataset, if ionophore use (non-medically important AMD) had been aggregated with category I through III AMU (medically important AMD), AMU would have been nearly 10 times that which was reported without category IV AMD. This would have obvious implications to users of these data if AMU was to be compared among groups, with some groups including ionophores in aggregate summaries and others not. When only medically important AMD (both in-feed and individually dosed) were considered (Table 10), the preponderance of use (almost 90% of medically important AMD) was category III AMD (8). Category I AMD (fluoroquinolones and ceftiofur) represented only a small fraction (<1%) of the medically important AMD used in feedlots; in addition, all category I AMD were individually dosed, and their use decreased over time. This is an encouraging sign that current practices in the feedlot industry support good antimicrobial stewardship, in that AMD of lesser importance to human medicine are being selected when feasible and effective (7). However, macrolides (category II AMD) still comprised ~12% of use, and their use remained fairly consistent throughout the years, suggesting that continued focus on antimicrobial stewardship in this area is essential. Of note, the WHO classifies ceftiofur, fluoroquinolones, and macrolides all as highest priority—critically important antimicrobials (HP-CIA) (33), further underscoring the importance of stewardship in these classes of AMD supported by surveillance data like those presented in the current study.

This study also emphasized the importance of transparency in clarifying the metric used to report AMU, particularly in livestock, since metrics have not been well-standardized (30, 34). Figures 2A,B demonstrated the contrast of the gAMD and nADD metric in the specific case of macrolides, which have a relatively low mg/kg dosage and a relatively long duration of effect. In this context, employment of the gAMD metric would result in the interpretation that less macrolides were used in the population than if the nADD metric was used. If only AMU in the same class of antimicrobials was being evaluated in the same production class of animals, the choice of metric would be immaterial. However, if the intention is to compare AMU across classes of AMD (for example, comparing macrolide to tetracycline use) or among different sized animals, the gAMD metric is problematic. Furthermore, because many of the more medically important category AMD, such as cephalosporins and macrolides, have lower dosage per kg rates, emphasis on mg/kg reduction targets could inadvertently discourage appropriate stewardship (35). In summary, weight of AMD can be a useful, intuitive metric if comparing AMU of the same AMD type. If a denominator of biomass or number of animals at risk of exposure is employed, it can potentially be used for comparisons among populations or even across species, but limitations of the metrics must be recognized and transparently reported. It is particularly important in this context that the animal weight used to calculate biomass at risk of exposure is appropriate and standardized among different populations (36). Issues regarding consequences of choice of metrics are covered in more detail in the accompanying paper (37).

Consistent with the primary importance of BRD as a health concern in fed cattle (9), about 40% of in-feed AMU and the majority of individually dosed AMU was related to BRD. The preponderance of individually dosed AMU was for metaphylaxis, and the assessed risk level of the cattle for BRD appeared to have a marked influence on AMD choice for metaphylaxis, with HR cattle far more likely to be exposed to a macrolide for metaphylaxis and less likely to be exposed to a tetracycline than LR cattle, and vice versa for tetracyclines. This is not surprising as macrolides have previously been shown to be highly effective AMD for the prevention of BRD in cattle populations at HR of developing BRD, which influences protocols for AMU (15). It should be noted that because of the relatively larger numbers of LR cattle placed in the studied feedlots compared to HR cattle, tetracyclines were still the most-used AMD on an nADD basis for BRD metaphylaxis. The assessed BRD risk of the cattle also had a less marked influence on AMD choices for BRD treatment. If protocols were unchanged, decreasing the proportions of HR cattle admitted to feedlots could reduce category II AMD (macrolide) and increase category III (tetracycline) use for metaphylaxis, which could be favorable from an AMD stewardship standpoint. However, some factors likely influencing the designation of cattle as HR for BRD, such as placement of cattle on feedlots during cold winter weather, would be difficult to modify given that one of the underlying reasons for placing the animals on the feedlot during this season is lack of winter pasture. Further, complicated questions about the economics of conditioning animals to reduce BRD risk (e.g., pre-feedlot vaccination, additional “backgrounding” time) have not, as of yet, been addressed within the current farm to slaughter beef industry continuum.

Overall use of AMD decreased over time throughout the study. Since in-feed tetracyclines made up the bulk of medically important AMU, the decrease in overall tetracycline use was primarily driven by the in-feed reduction observed for the indication of histophilosis therapy. This observation provided an interesting example of ability to use these AMU data to assess an intervention. Multi-year clinical studies performed by Feedlot Health just prior to the initiation of data collection for this study indicated that targeted parenteral metaphylaxis reduced the need for in-feed chlortetracycline to prevent and control histophilosis in specific populations. Implementation of new protocols drawing from this study likely resulted in the observed reduction of tetracycline use in-feed. Regarding observed trends for parenterally administered drugs, the overall reduction in AMU over time is most likely a result of continued efforts to improve animal health and welfare in a cost-effective manner. These could include changes regarding vaccine use, biosecurity, animal husbandry, detection of sick animals, metaphylaxis and treatment protocols, and risk assessment/assignment algorithms. The slight increase in the amount of individually dosed macrolides seen from PC1 to PC4 may have been a result of bolstered individually-dosed metaphylaxis. One could argue that it is undesirable to increase category II use while category III use was reduced. However, the magnitude of the reduction in tetracyclines (174,539 nADD/100,000 cattle) was much greater than the small increase observed in macrolides (2,625 nADD/100,000), and group exposures were reduced, arguably improving stewardship.

The other protocol alteration reflected in these AMU data was the addition of a newly licensed product combination of florfenicol and flunixin meglumine based on Feedlot Health clinical research (38) in the fall of 2008. The doubling in florfenicol use from PC1 to PC4 is explained by this protocol change and provides another interesting example of how detailed AMU data such as these could be used to assess effects of interventions.

Overall, this study demonstrated the importance of collecting farm level data to provide a comprehensive picture of AMU in the context of indication for use and animal characteristics. While census data were collected in this study, this is not a practical approach for ongoing, sustainable monitoring of AMU in feedlots due to the time and resources required for data retrieval, collation and analysis. Therefore, future research should focus on appropriate sub-sampling methods for representative monitoring of AMU in fed cattle.

## Data Availability Statement

The datasets generated for this study are available on request to the corresponding author.

## Ethics Statement

The protocol for this project was reviewed and approved by the Feedlot Health Management Services Ltd. Animal Care Committee (a certified holder of a Certificate of Good Animal Practice) and in accordance with standards set by the Canadian Council of Animal Care.

## Author Contributions

SB performed summarization, analysis, interpretation of data, and drafted the manuscript. SH was the principal investigator of the study and was involved in study planning, AMU data collection, finalization of data compilation and verification, data summarization and interpretation, and manuscript revision. SG and PM were involved in study planning, interpretation of results, and manuscript revision. BW, JW, JS, and CW assisted in collection, compilation, and verification of data. SO contributed to interpretation of the results and participated in manuscript revision. CB was involved in study planning, oversaw final data verification, summarization and interpretation of findings, and participated in manuscript revision.

### Conflict of Interest

CB is part owner and managing partner of Feedlot Health Management Services Ltd. and Southern Alberta Veterinary Services. SH, BW, JW, JS, and CW are employees at Feedlot Health Management Services Ltd., Okotoks, Alberta, Canada. Feedlot Health is a private company that provides expert consultation regarding production and management of feedlot cattle and calf grower calves, including developing veterinary protocols to support animal health. They also conduct in-house and contract research related to dairy calf grower and feedlot production. The remaining authors declare that the research was conducted in the absence of any commercial or financial relationships that could be construed as a potential conflict of interest.
